# Supplementary material for: Cathepsin K inhibitors promote osteoclast-osteoblast communication and engagement of osteogenesis
Source: JBMR Plus. 2025 May 6;9(8):ziaf079. doi: 10.1093/jbmrpl/ziaf079 (PMC12266958; doi:10.1093/jbmrpl/ziaf079)
Supplement: 27_April_2025_supplementary_file_JBMR_plus_ziaf079 [file 27_april_2025_supplementary_file_jbmr_plus_ziaf079.docx]

**Supplemetary material**

**Supplementary Figure S1**

**
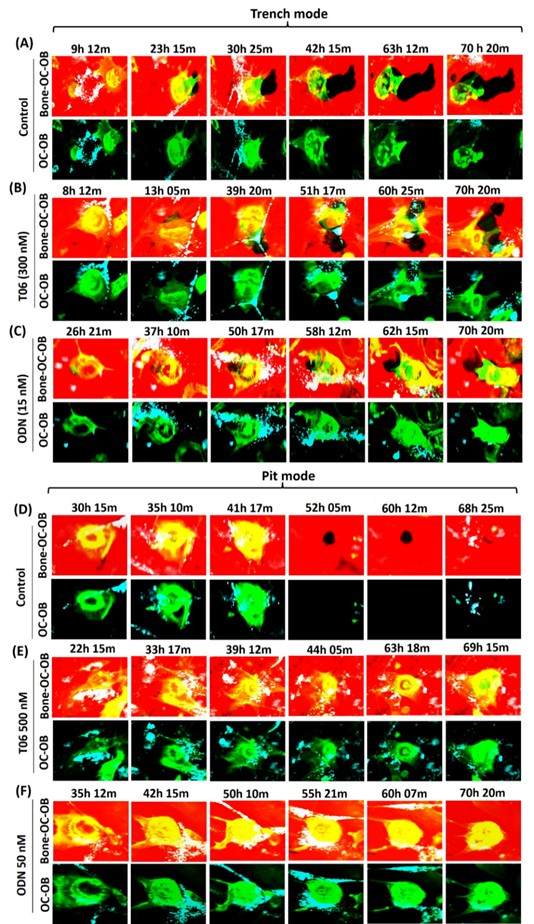
**

**Supplementary Figure S1: Effect of CatK inhibitors at low concentration on the association of OBs with trenches**. (A-C) Selected time-lapse images of OC-OB interaction during trench formation were taken from movies of control (A), T06 300 nM (B), ODN 15 nM (C) (video 2) at various time points over 72 h co-culture. OCs make long trenches in untreated conditions; however, dose dependent inhibition of CatK in OCs showed gradual decrease in size of trenches. OBs showed their preference to excavations generated by CatK-inhibited OCs compared to trenches generated under untreated conditions. (D-E) Effect of CatK inhibitors at high concentration on the association of OBs with pits. Selected time-lapse images of OC-OB interaction during pit formation were taken from videos of control (D), T06 500 nM (E), and ODN 50 nM (F) (video 3) at various time points over 72 h of co-culture. OBs showed their preference to pits generated by OCs subjected to CatK inhibitors (both T06 and ODN) compared to the pits generated under untreated conditions. OCs were stained for actin by using phalloidin (green), OBs were stained with Vybrant DiO cell-labeling solution (cyan) and bone surface was stained with rhodamine (red).

**Supplementary Figure S2**


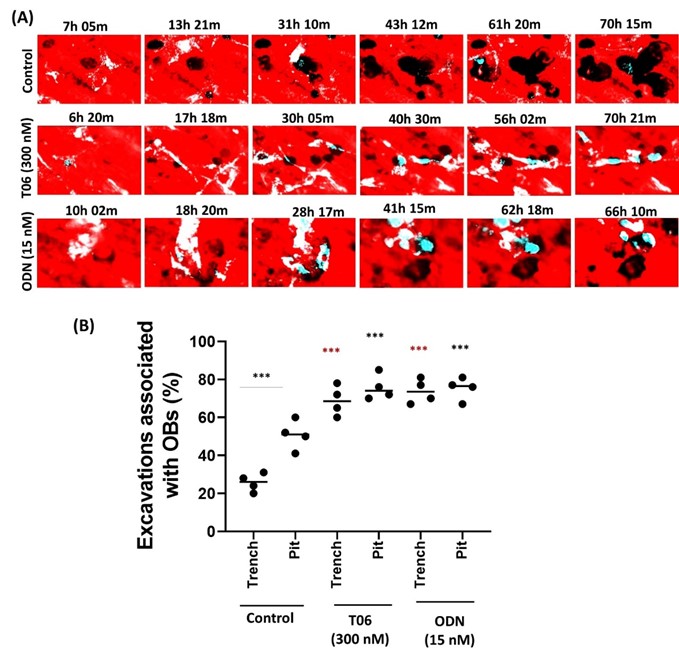


**Supplementary Figure S2:** **Effect of CatK inhibitors on the association of OBs with resorption events:** (A) Time-lapse images showed the interaction of OBs with the excavations in control, T06 (300 nM), and ODN (15 nM) conditions displayed OBs preference towards pits and this interaction increased with CatK inhibition **(supporting data for Fig. 2,3, and 4 and relevant videos; supplementary video 1)**. Sample size: Control: n= 4 donors; 300 nM T06: n=4 donors; 15 nM ODN: n=4 donors). For each donor, 2-3 replicate experiments on individual bone slices were analyzed for all conditions. The median obtained in each experiment are shown as bars. We analyzed between 40-120 OC-OB activities per condition per experiment for each of the donors). (B) Quantification of OB interaction with resorbed cavities under control and inhibitor treated condition. Statistics: Mann-Whitney test (***P < 0.001) to compare the OB interaction with pits or trenches under untreated control condition. Kruskal–Wallis’s test, two tailed (ns: not significant; ***P < 0.0001); Dunn’s multiple comparisons test ***P < 0.001) compared to control. Pits statistics are shown in black and trenches comparison is highlight in red asterisk.

**Supplementary Figure S3**


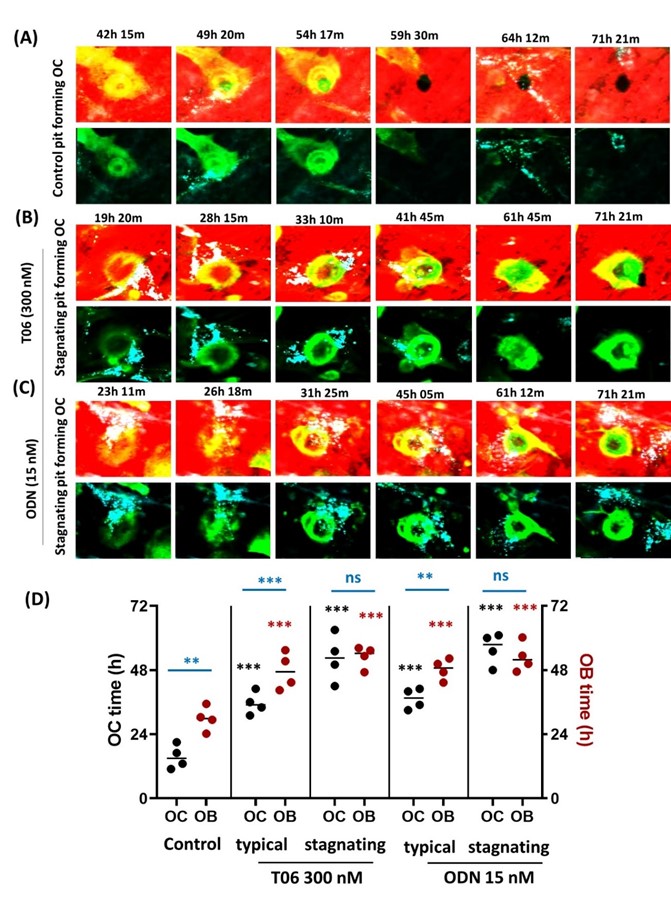


**Supplementary Figure S3: Induction of a stagnating activity state in OCs treated with CatK inhibitors and its consequence for duration of occupancy of the excavation by OCs and OBs.** Time-lapse images of cocultures showing the typical resorption behaviour of OCs in generating pit under (A) control, and atypical resorption behavior (B) T06 500nM, and (C) ODN 15 nM treated conditions during a 72 h observation. (D) Duration of presence of OCs and OBs when comparing typical behaviour, stagnating behaviour, and absence of treatment (video 4). Control: n= 4 donors; 300 nM T06: n=4 donors; 15 nM ODN: n=4 donors. For each donor, 2 replicate experiments on individual bone slices were analyzed for all conditions. The median obtained in each experiment are shown as bars. We analyzed between 40-120 OC-OB activities per condition per experiment for each of the donors). Statistics: Kruskal–Wallis’s test, two tailed (ns: not significant; **P < 0.01; ***P < 0.001); Dunn’s multiple comparisons test (ns: not significant; **P < 0.02; ***P < 0.001) compared to the untreated control (OC time: black stars; and OB time red stars). OBs recruitment in pits is also independent of OCs and OBs reside longer duration in pits compared to OCs in both untreated and treated conditions. Statistics: Mann-Whitney test (ns: not significant; **P < 0.01; ***P < 0.001) shown by blue stars.

**Supplementary Figure S4**


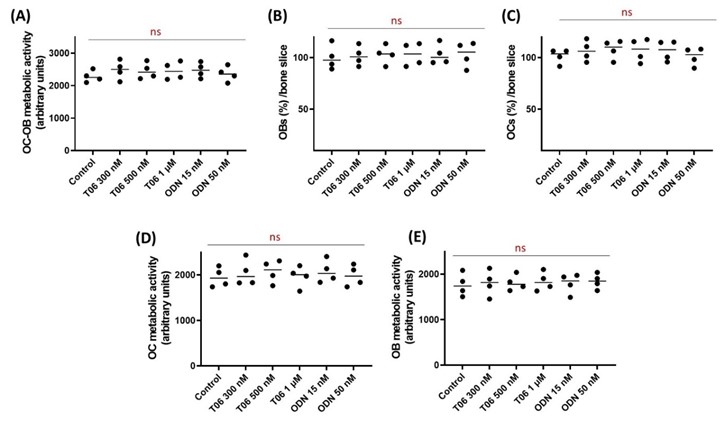


**Supplementary Figure S4:** Quantification of (A) metabolic activity, (B) ALP^+^ OBs, and (C)TRACP^+^ OCs in co-culture system grown over bone slice for 72 h in the presence or absence of inhibitors (T06, ODN at different concentrations). Quantification of metabolic activity of (D) OBs, and (E) OCs in mono-culture grown over bone surface to determine the effect of inhibitors (T06, ODN) on the cell viability and proliferation. Sample size (n=4 donors) for control, T06 (300 nM, 500 nM, 1 µM), ODN (15 nM, 50 nM). These data confirm no effect of inhibitors on cells number, viability and proliferation as no significant difference was observed compared to untreated control. Statistics: Kruskal–Wallis’s test, two tailed (ns: not significant) compared with untreated control.

**Supplementary Figure S5**


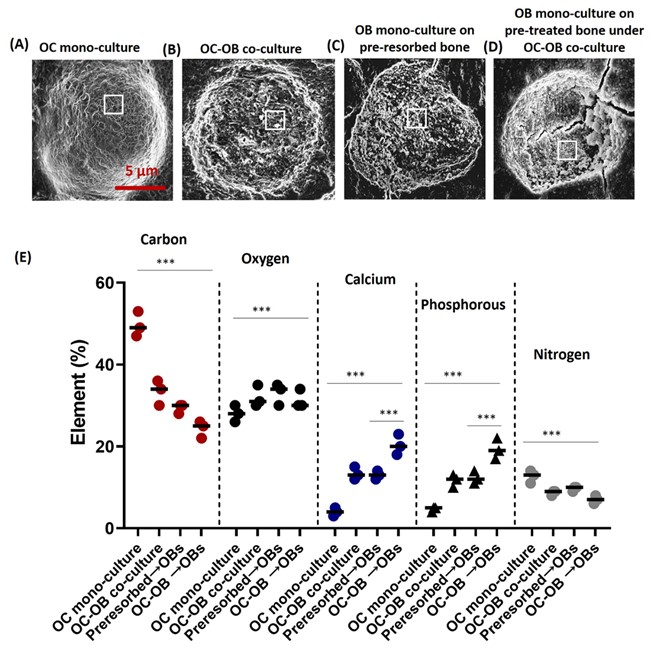


**Supplementary Figure S5: Effect of OBs on the ultrastructure and elemental composition of the resorption pits.** (A-C) SEM micrographs (backscattered electron imaging) clearly display the differences in matrix in pits after (A) an OC mono-culture. (B) an OC-OB co-culture, and (C) an OB-culture on pre-resorbed bone slices in co-culture media (D) an OB culture on pre-resorbed bone slice under OC-OB culture in co-culture media. Note the changes in the appearance of the matrix. Scale bar: 5 µm for pits in each condition. (E) Quantification of carbon, oxygen, calcium, phosphorous, and nitrogen in the pits under mono-culture, co-culture, and on pre-resorbed conditions. (5µm^2^ region of interest (ROI); 5-10 regions per pit in each condition for 20 pits). n=3 individual experiments from separate donors. The mean proportions obtained in each experiment are shown as bars. Statistics: Kruskal–Wallis’s test, two tailed (ns: not significant; *P = 0.05, ***P < 0.001); Dunn’s multiple comparisons test (ns: not significant; *P = 0.052, ***P < 0.001) compared with untreated control condition. OC-OB co-culture and OBs cultured on pre-resorbed bone slices showed no significant difference in element accumulation.

**Supplementary Figure S6**


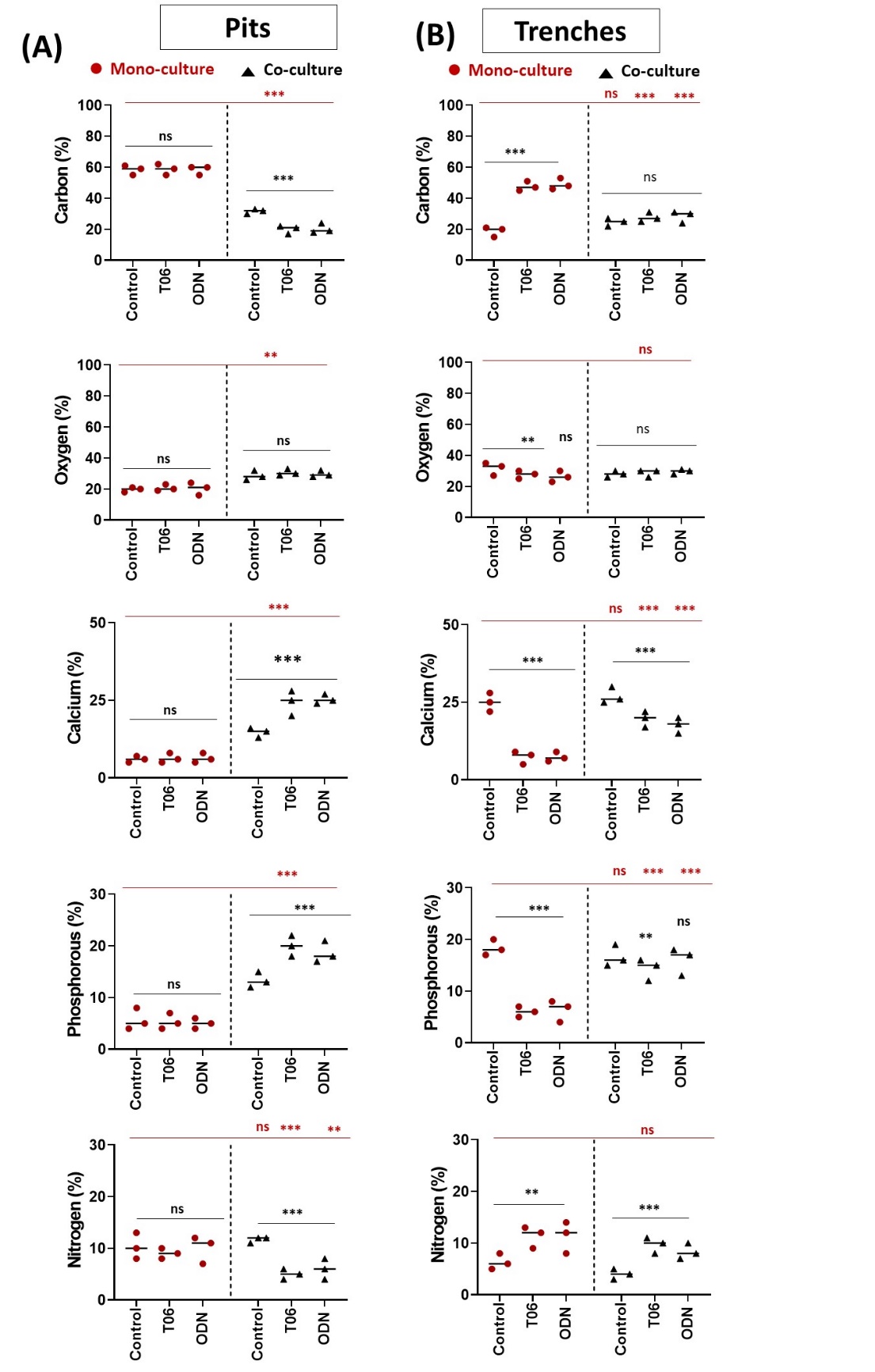


**Supplementary Figure S6: Quantification (atomic percentage) of carbon, oxygen, calcium, phosphorous, and nitrogen in (A) pits and (B) trenches generated in mono-culture and co-cultures with or without CatK inhibitors**. The elements were quantified by using energy dispersive X-ray spectroscopy. (Spectrum within 5µm^2^ region of interest (ROI); 5-10 regions per pit in each condition for 25 pits or trenches; n=3 individual experiments from separate donors). The median obtained in each experiment are shown as bars. Statistics: Kruskal–Wallis’s test, two tailed (ns: not significant; **P = 0.01, ***P < 0.001); Dunn’s multiple comparisons test (ns: not significant; **P = 0.008, ***P < 0.001) used to compare the treated conditions with untreated control within mono and co-culture (indicated by black asterisk). Mann–Whitney test was used to compare the elements of mono culture with the co-culture under control and inhibitor treated conditions (indicated by red asterisk). ‘ns’, not significant; **P < 0.01; ***P < 0.001.

**Supplementary Table S1: IC_50_ values of ODN and T06 for soluble collagen degradation and bone resorption in osteoclast assay.**

| **Compound name** | **Structure** | **Collagen degradation IC_50_ (μM)** | **Osteoclast assay IC_50_ (µM)** |
| --- | --- | --- | --- |
| Tanshinone IIA sulfonic sodium (T06) | 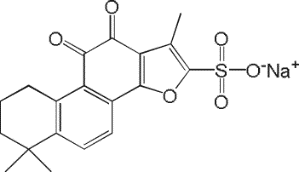 | 2.7 ± 0.2 | 0.24 ± 0.06 |
| Odanacatib  (ODN) | 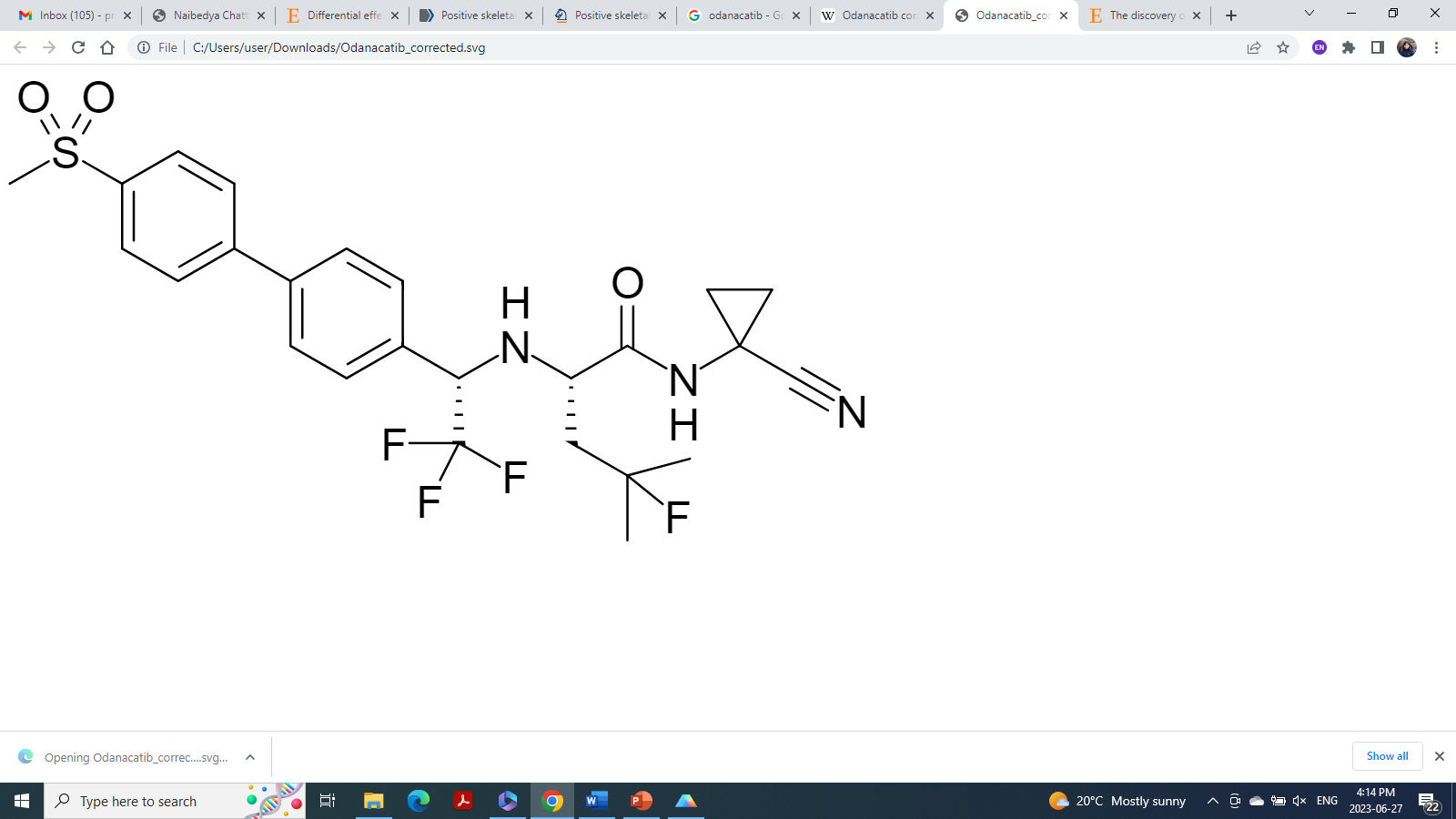 | 0.21 ± .05 | 0.014 ± 0.004 |

**Video captions**

**Video 1:** OB association with pit and trench resorption events: Osteoblast responses vary by resorption mode, with more sustained OB-OC interactions marked by higher occupancy and longer durations in pits compared to trenches.

**Video 2:** CatK inhibition strengthens OB association with trench resorption event: Low-dose T06 (300 nM) and ODN (15 nM) treatments allow OCs to form small trenches and increased pits. Interestingly, trench resorption events under these conditions display prolonged and frequent interactions with OBs compared to controls.

**Video 3:** CatK inhibition strengthens OB association with pits: Maximum CatK inhibition with T06 (1 µM) and ODN (50 nM) prevents the switch to trench mode, resulting in predominant pit formation. Under these conditions, OCs remain engaged longer in pit formation, correlating with extended OB interactions.

**Video 4:** OBs remain closely associated with “stagnating” OCs for as long as the OCs occupy the resorption cavity: CatK inhibition promotes this stagnation in OCs co-cultured with OBs, extending their time within pits an effect that is further amplified during periods of stagnating activity.
